# What is an acceptably smoothed fluence? Dosimetric and delivery considerations for dynamic sliding window IMRT

**DOI:** 10.1186/1748-717X-2-42

**Published:** 2007-11-23

**Authors:** Nicolini Giorgia, Fogliata Antonella, Vanetti Eugenio, Clivio Alessandro, Ammazzalorso Filippo, Cozzi Luca

**Affiliations:** 1 Oncology Institute of Southern Switzerland, Medical Physics Unit, Bellinzona, Switzerland; 2 Medical Physics Specialisation School, University of Milan, Milan, Italy; 3 Faculty of Medicine, University of Lausanne, Lausanne, Switzerland; 4 Biomedical Physics, Radiooncology Dept, Uniklinik für Radioonkologie Tübingen, Tübingen, Germany

## Abstract

**Background:**

The study summarised in this report aimed to investigate the interplay between fluence complexity, dose calculation algorithms, dose calculation spatial resolution and delivery characteristics (monitor units, effective field width and dose delivery against dose prediction agreement) was investigated. A sample set of complex planning cases was selected and tested using a commercial treatment planning system capable of inverse optimisation and equipped with tools to tune fluence smoothness.

**Methods:**

A set of increasingly smoothed fluence patterns was correlated to a generalised expression of the Modulation Index (MI) concept, in nature independent from the specific planning system used that could therefore be recommended as a predictor to score fluence "quality" at a very early stage of the IMRT QA process. Fluence complexity was also correlated to delivery accuracy and characteristics in terms of number of MU, dynamic window width and agreement between calculation and measurement (expressed as percentage of field area with a *γ *> 1 (%FA)) when comparing calculated vs. delivered modulated dose maps. Different resolutions of the calculation grid and different photon dose algorithms (pencil beam and anisotropic analytical algorithm) were used for the investigations.

**Results and Conclusion:**

i) MI can be used as a reliable parameter to test different approaches/algorithms to smooth fluences implemented in a TPS, and to identify the preferable default values for the smoothing parameters if appropriate tools are implemented; ii) a MI threshold set at MI < 19 could ensure that the planned beams are safely and accurately delivered within stringent quality criteria; iii) a reduction in fluence complexity is strictly correlated to a corresponding reduction in MUs, as well as to a decrease of the average sliding window width (for dynamic IMRT delivery); iv) a smoother fluence results in a reduction of dose in the healthy tissue with a potentially relevant clinical benefit; v) increasing the smoothing parameter s, MI decreases with %FA: fluence complexity has a significant impact on the accuracy of delivery and the agreement between calculation and measurements improves with the advanced algorithms.

## Background

Intensity modulated radiation therapy (IMRT) is known to improve the conformal avoidance in external beam radiotherapy. Literature offers a huge variety of studies, at planning or clinical level, where a plethora of inverse planning algorithms have been investigated [1-5] to explore IMRT performances under several points of view.

The optimisation process is a computational problem, potentially susceptible to noise and artefacts (high frequency spatial fluctuations) producing sharp fluence peaks and valleys in millimetric spatial scale. These features could translate into difficult patterns for the delivery system, prolonged beam-on time and increased sensitivity to all conventional treatment uncertainties. Mohan et al [6] used the term "complexity" to describe the frequency and amplitude of fluctuations in the intensity distribution of a beam. The authors demonstrated that, as a trade-off, the more 'complex' the intensity patterns, the higher the number of monitor units (MU) will be to deliver the prescribed doses. This could affect, due to linac potential limitations, both the quality and accuracy of delivered doses.

Several authors suggested as a recommended solution to systematically adopt planning tools and methods able to optimise smooth beam fluences [7-15]; Coselman et al [16] underlined that smoothing algorithms that are applied post-optimisation, usually result in a degradation of the plan according to the objective function while, when the smoothing is part of the objective function, better results are obtainable.

In 2001 Webb [17] suggested the use, in the optimisation process, of a cost function that included two special terms: one accounting for the fluence changes in adjacent pixels (bixels in Webb's study), and the second one related to the minimum allowed field size to minimise unwanted consequences of a high degree of modulation.

Fluence complexity is also strongly interconnected to the quality and efficiency of dose delivery (and consequently also to radiation protection issues). The first aspect relates to the capability of linear accelerators and multileaf collimators to generate complicate dose patterns, the second relates to the time (and MU) needed to deliver those patterns.

Webb proposed [18], as a general rule of thumb for good IMRT practice, to avoid excessive complexity and, as a metric to appraise the degree of modulation in a fluence matrix, introduced the concept of Modulation Index, MI, [19]. This metric was already used by our group in a previous study [20] to investigate potential differences between static and dynamic IMRT delivery with the sliding window method. The present study was conceived to further analyse if the MI can be prospectively used to discriminate between acceptable, questionable and necessary fluences.

Possible correlations between MI and fluence smoothing parameters, dose calculation algorithms, dose calculation spatial resolution and delivery characteristics (MU, effective field width and dose delivery against dose prediction agreement) were investigated using the commercial treatment planning system (TPS) Eclipse, Varian to test the potential clinical impact of the fluence modulation degree.

## Methods

**Figure 1:**
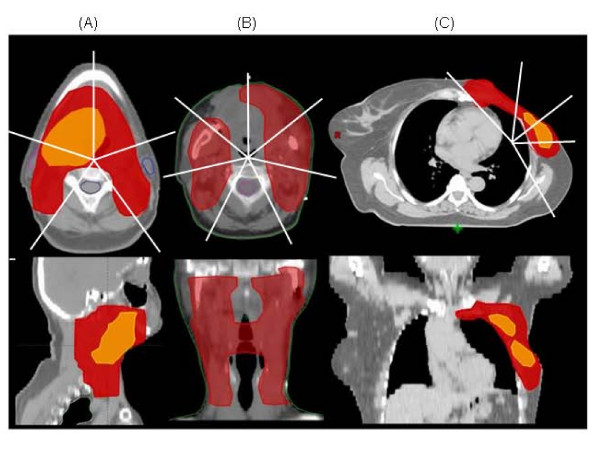
The three planning cases selected for the study. (A) base of tongue, (B) mandible, (C) left thoracic wal. An axial CT slice approximately at the centre of the target volume is shown together with a reconstructed coronal or sagittal view; target volumes are shown as overlays.

Three different IMRT planning cases (two head and neck and one breast) were selected as representative of demanding planning requirements. Table 1 provides some information on the selected test cases. Two of the cases were to be planned for simultaneous integrated boost (SIB) with two dose levels (1.8 and 2.2 Gy per fraction in 30 fractions) and one case presented a very irregular target shape. Figure 1 shows target volumes overlaid to the CT data in axial and in sagittal or coronal views. Lines represent the beam directions used to optimise the dose plans and give a general overview on the beam ballistic and techniques used. All beams were coplanar. Plans were designed using the Eclipse TPS from Varian (release 7.3.10) and its inverse Dose Volume Optimizer (DVO, vers. 7.5.14.3) [21-25] for delivery according to the dynamic sliding window method.

**Table 1:** Summary of indications, dose prescriptions and volumes of interest for the three planning cases selected for the study.

	Case 1	Case 2	Case 3
Site	Base of tongue	Mandible	Left Thoracic wall
Dose per fraction [Gy]	2.2/1.8 *	1.8	2.18/2.00*
Number of fractions	30	27	25
Number of fields (splitted)	5 (4)	7 (7)	5 (4)
PTV(s) volume [cm^3^]	153/755	680	123/969
OARs	Healthy tissue, spinal cord, parotids	Healthy tissue, spinal cord, parotids	Healthy tissue, heart, ipsi- and contra-lateral lung, homer, contra-lateral breast

* Simultaneous Integrated Boost

Plans were developed for a 6 MV photon beam from a Varian Clinac 6EX equipped with a 120 leaves MLC. Numerical parameters relevant for dynamic delivery of MLC were set in Eclipse as follows: leaf transmission: 1.8%; dosimetric leaf gap: 2.3 mm; minimum dose dynamic leaf gap 0.6 mm; dose dynamic leaf tolerance: 2 mm; dose rate: 300 MU/min. Leaf sequencing and delivery are based on the dynamic sliding window technique.

In Eclipse, optimal fluence smoothing is part of the DVO algorithm and it is performed along two directions: X, parallel to the MLC movement and Y, orthogonal to it. Smoothing is applied at each optimisation iteration by adding two smoothness-related planning objectives in the cost function that account for the difference between neighbouring fluence values. The objective function becomes:

F(x)=∑iwi(Di−Pi)2+∑kwk(xk+1−xk)2
 MathType@MTEF@5@5@+=feaafiart1ev1aaatCvAUfKttLearuWrP9MDH5MBPbIqV92AaeXatLxBI9gBaebbnrfifHhDYfgasaacPC6xNi=xI8qiVKYPFjYdHaVhbbf9v8qqaqFr0xc9vqFj0dXdbba91qpepeI8k8fiI+fsY=rqGqVepae9pg0db9vqaiVgFr0xfr=xfr=xc9adbaqaaeGacaGaaiaabeqaaeqabiWaaaGcbaqaaiabdAeagjabcIcaOiabdIha4jabcMcaPiabg2da9maaqafabaGaem4DaC3aaSbaaSqaaiabdMgaPbqabaGccqGGOaakcqWGebardaWgaaWcbaGaemyAaKgabeaakiabgkHiTiabdcfaqnaaBaaaleaacqWGPbqAaeqaaOGaeiykaKYaaWbaaSqabeaacqqGYaGmaaGccqGHRaWkdaaeqbqaaiabdEha3naaBaaaleaacqWGRbWAaeqaaOGaeiikaGIaemiEaG3aaSbaaSqaaiabdUgaRjabgUcaRiabbgdaXaqabaGccqGHsislcqWG4baEdaWgaaWcbaGaem4AaSgabeaakiabcMcaPmaaCaaaleqabaGaeeOmaidaaaqaaiabdUgaRbqab0GaeyyeIuoaaSqaaiabdMgaPbqab0GaeyyeIuoaaaaa@540E@

where the first addendum is the usual component for dose-volume constraints: *P*_*i *_is the prescribed doses per each volume voxel *i *while *D*_*i *_is the dose computed at point *i *and expressed as *D*_*i *_= *d*_1, *i*_*x*_1 _+ *d*_2, *i*_*x*_2 _+...+ *d*_*J*, *i*_*x*_*J *_where *x*_*j *_is the weight of the *j*^*th *^beamlet in the fluence map and *d*_*j*, *i *_is the dose to point *i *from the *j*^*th *^beamlet (i.e. dose to point *i *is a weighted sum of the dose from all beamlets).

The second addendum is related to the smoothing, and operates on the beamlet weighting factors aiming to reduce large steps between neighbouring beamlets. The two weights *w*_*k *_(X- and Y-Smooth parameters in the following) are adjustable by users during the plan optimisation phase and regulate the importance of the smoothing component in the gradient search.

To appraise the effectiveness of fluence smoothing and its interplay with other planning variables, the study was organised performing full optimisation and dose calculation for all the combinations of the following three variables:

• *Smoothing parameters*: X- and Y- Smooth described above, s in the following, were varied simultaneously and set to 25, 50 and 80 (s25, s50 and s80 in the following) being the higher the values the higher the smoothing of the fluence patterns. Routinely, in clinical practice, X- and Y- Smooth are set in the range 30–100. In general, more emphasis is required in smoothing the fluence in the X direction, to minimise MUs, rather then in the Y direction but, from the accuracy of delivery point of view, both directions have the same relevance.

• *Dose calculation algorithm*: two algorithms were used: the Single Pencil Beam Algorithm (PBC), and the newly introduced convolution/superposition algorithm Anisotropic Analytical Algorithm (AAA) [26-28].

• *Spatial resolution of dose calculation matrix*: two grids were used: 2.5 (the minimum grid for PBC) and 5 mm. 2.5 mm is also the internal grid size used by Eclipse to compute and store fluences.

For each experiment (a combination of the three above variables, for a total of 12 experiments) optimisation was carried out using a fixed set of dose volume objectives. For each experiment 17 modulated beams (two 5-field and one 7-field plans) were obtained from the dose plans. A total of 36 dose plans and 204 modulated beams were compared and fed into the analysis process.

The analysis was stratified at multiple levels. A spectral analysis was performed to appraise general characteristics of the fluence patterns and to derive a single predictive parameter representing the fluence complexity. Dose volume histograms and MU were analysed to identify (if any) potential direct correlation between fluence complexity and dose distribution quality from a clinical perspective. Pre-treatment verification measurement were finally used to identify the impact of fluence complexity on delivery vs calculation agreement and to validate the predictive power of the parameter derived form the spectral analysis.

### Spectral analysis and Modulation Index

The degree of fluence modulation was studied analysing the spectrum and the derived Modulation Index (MI), concepts introduced by Webb [19].

The definition of the spectrum and its calculation, that was originally defined in one dimension, was here generalised accounting for intensity value changes along both X and Y directions, and along the X-Y diagonals, generating directly a mean spectrum for the whole fluence matrix.

The spectrum *Z*, therefore, is obtained as the average of three components:

*Z*(*f*) = [*Z*_*x*_(*f*) + *Z*_*y*_(*f*) + *Z*_*xy*_(*f*)]/3

where, considering an intensity fluence map *I*_*i*, *j *_of size n × m,

Zx(f)=1n(m−1)Nx(f;Δx>fσI)
 MathType@MTEF@5@5@+=feaafiart1ev1aaatCvAUfKttLearuWrP9MDH5MBPbIqV92AaeXatLxBI9gBaebbnrfifHhDYfgasaacPC6xNi=xI8qiVKYPFjYdHaVhbbf9v8qqaqFr0xc9vqFj0dXdbba91qpepeI8k8fiI+fsY=rqGqVepae9pg0db9vqaiVgFr0xfr=xfr=xc9adbaqaaeGacaGaaiaabeqaaeqabiWaaaGcbaqbaeqabeGaaaqaaiabdQfaAnaaBaaaleaacqWG4baEaeqaaOGaeiikaGIaemOzayMaeiykaKIaeyypa0tcfayaamaalaaabaGaeeymaedabaGaeeOBa4MaeiikaGIaeeyBa0MaeyOeI0IaeeymaeJaeiykaKcaaaaakeaacqWGobGtdaWgaaWcbaGaemiEaGhabeaakiabcIcaOiabdAgaMjabcUda7iabfs5aejabdIha4jabg6da+iabdAgaMHGaciab=n8aZnaaBaaaleaacqqGjbqsaeqaaOGaeiykaKcaaaaa@4A33@

Zy(f)=1(n−1)(m)Ny(f;Δy>fσI)
 MathType@MTEF@5@5@+=feaafiart1ev1aaatCvAUfKttLearuWrP9MDH5MBPbIqV92AaeXatLxBI9gBaebbnrfifHhDYfgasaacPC6xNi=xI8qiVKYPFjYdHaVhbbf9v8qqaqFr0xc9vqFj0dXdbba91qpepeI8k8fiI+fsY=rqGqVepae9pg0db9vqaiVgFr0xfr=xfr=xc9adbaqaaeGacaGaaiaabeqaaeqabiWaaaGcbaqbaeqabeGaaaqaaiabdQfaAnaaBaaaleaacqWG5bqEaeqaaOGaeiikaGIaemOzayMaeiykaKIaeyypa0tcfayaamaalaaabaGaeeymaedabaGaeiikaGIaeeOBa4MaeyOeI0IaeGymaeJaeiykaKIaeiikaGIaeeyBa0MaeiykaKcaaaaakeaacqWGobGtdaWgaaWcbaGaemyEaKhabeaakiabcIcaOiabdAgaMjabcUda7iabfs5aejabdMha5jabg6da+iabdAgaMHGaciab=n8aZnaaBaaaleaacqqGjbqsaeqaaOGaeiykaKcaaaaa@4BF2@

Zxy(f)=1(n−1)(m−1)Nxy(f;Δxy>fσI)
 MathType@MTEF@5@5@+=feaafiart1ev1aaatCvAUfKttLearuWrP9MDH5MBPbIqV92AaeXatLxBI9gBaebbnrfifHhDYfgasaacPC6xNi=xI8qiVKYPFjYdHaVhbbf9v8qqaqFr0xc9vqFj0dXdbba91qpepeI8k8fiI+fsY=rqGqVepae9pg0db9vqaiVgFr0xfr=xfr=xc9adbaqaaeGacaGaaiaabeqaaeqabiWaaaGcbaqbaeqabeGaaaqaaiabdQfaAnaaBaaaleaacqWG4baEcqWG5bqEaeqaaOGaeiikaGIaemOzayMaeiykaKIaeyypa0tcfayaamaalaaabaGaeeymaedabaGaeiikaGIaeeOBa4MaeyOeI0IaeGymaeJaeiykaKIaeiikaGIaeeyBa0MaeyOeI0IaeGymaeJaeiykaKcaaaaakeaacqWGobGtdaWgaaWcbaGaemiEaGNaemyEaKhabeaakiabcIcaOiabdAgaMjabcUda7iabfs5aejabdIha4jabdMha5jabg6da+iabdAgaMHGaciab=n8aZnaaBaaaleaacqqGjbqsaeqaaOGaeiykaKcaaaaa@523A@

*N *is the number of changes for which

*N*_*x*_: Δ*x *= abs(*I*_*i*, *j *_- *I*_*i *+ 1, *j*_) > *f σ*_I_, with *i *= 1 to n-1, and *j *= 1 to m

*N*_*y*_: Δ*y *= abs(*I*_*i*, *j *_- *I*_*i*, *j *+ 1_) > *f σ*_I_, with *i *= 1 to n, and *j *= 1 to (m-1)

*N*_*xy*_: Δ*xy *= abs(*I*_*i*, *j *_- *I*_*i *+ 1, *j *+ 1_) > *f σ*_I_, with *i *= 1 to n-1, and *j *= 1 to (m-1)

*f *= 0.01,0.02,...,2 and *σ*_I _is the standard deviation (SD) of the submatrix *I*(*i*: *i *+ 1, 1: *j*).

Hence, *Z*(*f*) is the fraction of changes among adjacent bixels (in the two-dimensional frame) that exceed a certain fraction (*f*) of the SD.

For each fluence map, as a measure of the degree of modulation, the Modulation Index, MI, has been computed according to:

MI(F)=∫0FZ(f)df
 MathType@MTEF@5@5@+=feaafiart1ev1aaatCvAUfKttLearuWrP9MDH5MBPbIqV92AaeXatLxBI9gBaebbnrfifHhDYfgasaacPC6xNi=xH8viVGI8Gi=hEeeu0xXdbba9frFj0xb9qqpG0dXdb9aspeI8k8fiI+fsY=rqGqVepae9pg0db9vqaiVgFr0xfr=xfr=xc9adbaqaaeGacaGaaiaabeqaaeqabiWaaaGcbaGaemyta0KaemysaKKaeiikaGIaemOrayKaeiykaKIaeyypa0Zaa8qmaeaacqWGAbGwcqGGOaakcqWGMbGzcqGGPaqkcqWGKbazcqWGMbGzaSqaaiabicdaWaqaaiabdAeagbqdcqGHRiI8aaaa@3CF4@ with *F *= {0.1, 0.3,0.5, 0.6,0.8,1.0}

The integration limit F, which has no specific meaning in the conceptual definition of MI, was varied to appraise its dependence from the various computational conditions and to select, a posteriori, its best value for the purpose.

Spectrum and MI are, by definition, independent from the dose calculation algorithm and the spatial resolution of the dose computation grid.

### Dose calculation and clinical impact (DVH and MU)

For each value of the smoothing parameter s, four 3D dose distributions on patient's CT data were computed as described above by changing calculation algorithm (PBC and AAA) and dose calculation grid (2.5 mm and 5.0 mm). Dose distributions were analysed in terms of DVH for the planning target volumes (PTV) and for the organs at risk (OAR). A set of standard physical quantities were considered: mean, D_X _(percentage dose received by at least X% of the volume) and V_Y _(volume receiving at least Y% of the prescribed dose). Maximum and minimum significant doses were defined, according to ICRU (reports 50 and 62), in a 'significant' region equivalent to a sphere of 1.8 cm^3 ^(radius 0.75 cm). Target dose homogeneity was expressed as (D_5_–D_95_).

MUs for each plan were recorded and reported as MU/Gy to directly relate to the time needed to deliver a treatment and to the intrinsic efficiency of the delivery process.

The average MLC aperture (computed from the MLC steering files) during the dynamic delivery was reported as an intuitive metric of delivery complexity and of potential dosimetric limitations (the narrower the worse).

### Pre-treatment verification and delivery reliability

Delivery reliability was investigated by means of standardised pre-treatment verification methods. All 204 modulated beams were processed according to the quality assurance procedures enforced in our institute. Pre-treatment dosimetric verification was performed, for all combinations of smoothing parameter, dose calculation algorithm and dose grid, with the methodology described in detail in [29]. Images acquired with the amorphous silicon Portal Vision PV-aS500 connected to the linac were converted into absorbed dose in water at the depth of d_max_, and compared to the dose matrices computed by Eclipse at the same depth in water. The evaluation was based on the Gamma Index (*γ*) analysis [30] with criteria of distance to agreement DTA = 3 mm and dose difference ΔD = 3%. The dose difference was computed with respect to the significant maximum of each field. The scoring parameter used for the analysis was the percentage of the field area defined by the jaws resulting with *γ *> 1 (%FA). The acceptability criteria adopted in our institution and derived from in-house statistics of QA finding are: values of %FA should be smaller than 5%; for %FA values between 5 and 10% further investigations are performed; for values larger than 10% a re-planning is recommended.

## Results

### Spectral analysis and Modulation Index

**Figure 2:**
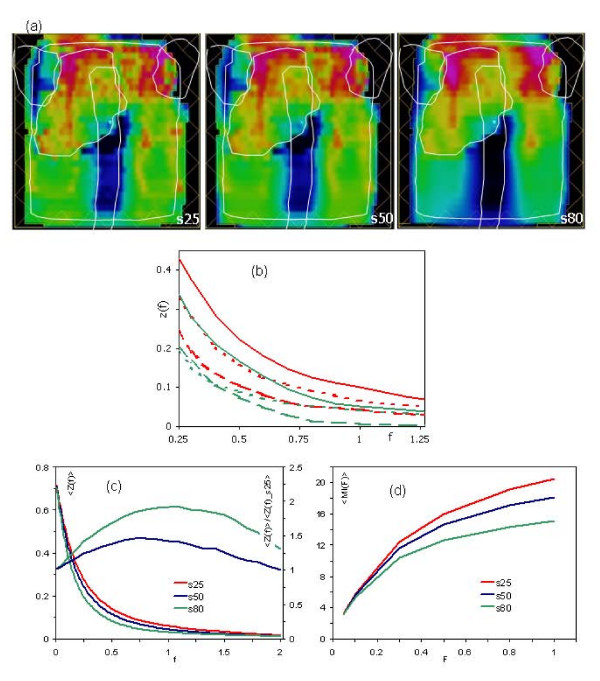
(a) Example of fluence, for a Head and Neck case, for the three smoothing conditions. The white overlays show the target volumes and the main organs at risk. (b) The three components *Z*_*x*_(*dotted lines*), *Z*_*y *_(dashed lines) and *Z*_*xy *_(solid lines) of the total spectrum for the two extreme conditions s25 (red) and s80 (green). (c) Mean spectra, averaged over the 17 fluence maps for the three smoothing conditions. It is shown also the ratio between the spectra for s50 (s80) and s25 respectively. (d) Mean modulation index MI.

Figure 2a shows the actual fluence matrix of one intensity modulated beam from the base of tongue planning case for the three smoothing conditions: s25, s50 and s80. Overlaid to the fluence matrix are the outlines of the two target volumes (SIB), spinal cord and parotids to better appraise the spatial distribution of fluence with respect to the clinical structures.

Figure 2b shows the three components *Z*_*x*_, *Z*_*y *_and *Z*_*xy *_of the total spectrum for the two extreme conditions s25 and s80. Of notice that the *Z*_*xy *_component is dominating over the other two. This is due to the fact that in the optimisation process, no smoothing is applied in this cross direction but the importance of x-y fluence discontinuities cannot be ignored when delivery accuracy issues are to be considered and 2D evaluations like the *γ *pass/fail analysis are performed. Results for all other fields and planning cases are consistent with the example shown. In Eclipse, differences between spectra obtained from actual and optimal fluences are negligible, as pointed out in [20].

Figure 2c presents the mean spectra, averaged over all the 17 actual fluence maps from the three planning cases for the three smoothing conditions. Curves never intersect, meaning that the smoothing tool in Eclipse is effective over the entire domain of fluence changes and that consistently, the higher the level of smoothing, the smaller the high frequency part of the spectrum will be. The values of the standard deviation SD, computed point by point on these mean spectra, are inversely proportional to the degree of smoothing. As an example, for f = 1, SD = 0.007 for s80 while SD = 0.014 (0.016) for s50 (s25) respectively. This result suggests that, since inter-beam spectral variability can be significantly reduced when sufficient smoothing is applied, as a consequence, a better uniformity in delivery accuracy and in MU/Gy calculation can be expected with clear potential benefits.

In Figure 2c is shown also the ratio between the spectra for s50 (s80) and s25 respectively. These ratios present a maximum value of 1.5 (1.9) for s50 (s80) for *f *~ 1. This result proves the fact expected from the smoothing concept that, in average, the s50 and s80 cases present maximum differences with respect to s25 in the range of moderate-high fluence changes (values of *f *around 1) between adjacent pixels.

Figure 2d shows, for s25, s50 and s80, the mean modulation index *MI*(*F*), averaged as described above, for various integration limits *F*. *MI *curves do not saturate but begin to flatten at *F *= 1 reflecting the previous result about the ratio of the spectra. The presence of a plateau (or the tendency to reach it) confirms that fluctuations in the spectra do not affect *MI *calculation and that *MI *could be used as a stable and reliable measure of the degree of modulation of an IMRT field. In the following, the integration limit of *F *= 1 was considered as the reference and results will be presented and discussed accordingly.

### Dose calculation: DVH and MU

**Figure 3:**
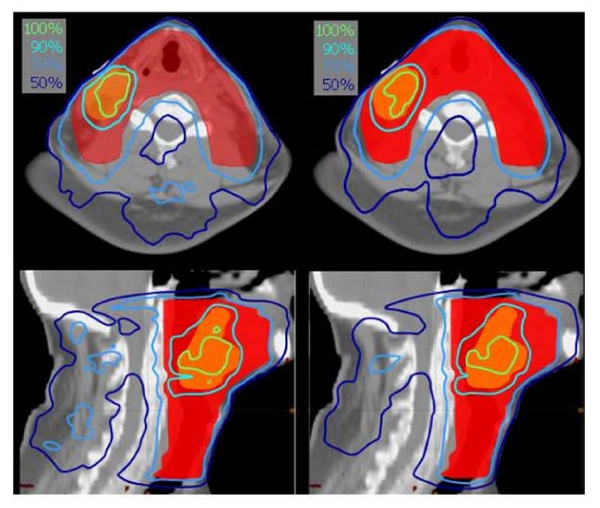
Examples of isodose distributions (Head and Neck case) for the two extreme smoothing conditions s25 (left) and s80 (right). Isodose lines are normalised to the dose prescribed to the smaller volume receiving the higher prescribed dose. The 73% isodose refers to the 90% of the dose prescribed to the large volume.

Figure 3 shows examples of isodose distributions for the base of tongue planning case relative to the s25 (left) and s80 (right) case. Dose distributions shown here are computed with the AAA algorithm and with a dose grid of 2.5 mm. Data are shown for an axial CT image containing the two target volumes (SIB) and for a sagittal view. Target volumes are overlaid as colour wash while isodoses are given by thick lines. Being the optimisation carried out for two dose levels, normalisation was set for the highest level and in the picture the 73% isodose line corresponds to the 90% of the dose prescribed to the large volume. In this way, for both target volumes the relative 90% isodose line is shown.

**Figure 4:**
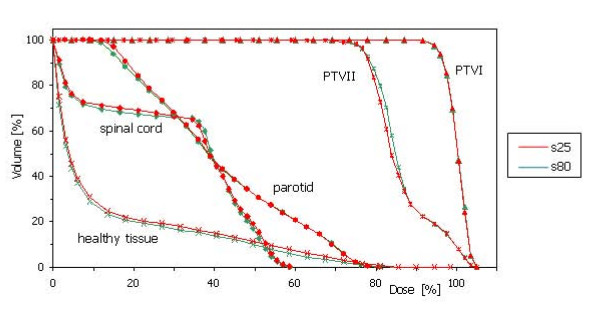
DVHs of targets and organs at risk (Head and Neck case) for the two extreme smoothing conditions s25 and s80.

Figure 4 shows DVH for the target volumes, spinal cord, parotids and healthy tissue for the s25 and s80 experiments.

No significant difference is present to allow different ranking of the two concurrent plans even if noise and ripples are visible on the isodose distributions. It is clear that, especially for regions out of the target (in this case posteriorly to the spinal cord) there is an over-modulation for s25 resulting in undesired isles of high dose or 'noisy' dose distributions.

A summary of the DVH analysis is reported in table 2. Mean values and standard deviations are given for some of the dose related quantities investigated in the study. Numbers refer to the differences between values obtained for the s80 and s25 experiments and are averaged over all target volumes and organs at risk. As for figure 4, data refer to the AAA dose calculation algorithm and to a calculation grid of 2.5 mm. Results do not change significantly if PBC or the coarser resolution or if the comparison is performed between s50 and s25. As it can be seen from the table, there is no significance in the difference (computed by means of two-sided paired t-test) between DVH related information from the s80 or the s25 simulations but, in general, DVH analysis is not particularly sensitive to noise in the dose distributions.

**Table 2:** DVH analysis: differences between plans obtained with s80 and s25. Reported are the mean, SD, range and p value. Data are averaged over the three planning cases, and reported for one dose calculation algorithm (AAA) and one dose grid (2.5 mm).

	Mean ± SD	Range	p
Targets			
Mean dose (%)	0.2 ± 0.3	[0.0, 0.5]	0.16
Min sign dose (%)	-0.4 ± 1.4	[-2.7, 0.8]	0.52
Max sign dose (%)	-0.4 ± 0.4	[-0.9, -0.1]	0.09
D_5_–D_95 _(%)	0.5 ± 0.8	[-0.2, 1.5]	0.24
Organs at Risk			
Mean dose (Gy)	-0.1 ± 0.9	[-0.8, 1.6]	0.77
Max sign dose (Gy)	-0.6 ± 1.4	[-3.3, 0.7]	0.35

**Figure 5:**
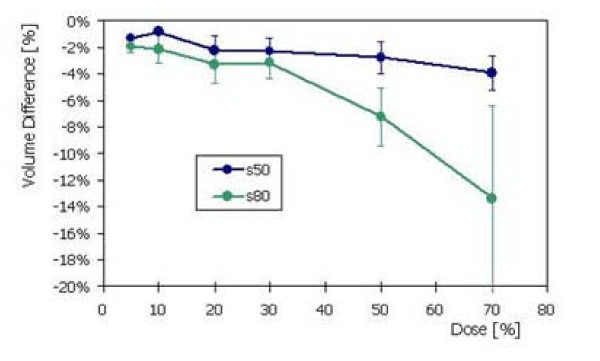
Healthy tissue analysis: volumetric differences for the s50 (or s80) plans and the s25 plans, as a function of different dose levels.

Figure 5 summarises findings observed for Healthy Tissue. The graph presents the differences of volume receiving at least a certain amount of prescribed dose for the s50 or s80 as compared to s25. Negative values mean that for s50 and s80 cases, the expected dose bath is systematically lower than for s25 (p < 0.05 for each of the three planning cases) with potential implications in terms of long term effects.

Table 3 summarizes results for the parameters directly linked to the efficiency of the delivery process. Data are reported as mean value and standard deviation averaging over the three planning cases and/or the 17 modulated beams; also the difference between observations for s25 and s80 cases are reported together with the corresponding p values computed with two-sided paired t-test. In the table, data are reported only for the AAA and for 2.5 mm. Results do not change significantly for the other configurations showing variations smaller than 1% for MU/Gy (for the other parameters the dose calculation algorithm is not relevant). To allow a direct comparison between fluence complexity and efficiency parameters, in table 3 average values of *MI *(integrated for *F *= 1) are similarly reported. A significant difference was observed with a reduction between 30% and 40% in MU/Gy or MLC average aperture when changing smoothing from s25 to 80.

**Table 3:** Summary of parameters linked to the efficiency of the delivery process. Data are reported as mean value and standard deviation averaging over the three planning cases and/or the 17 modulated beams. Data refer to AAA dose calculation algorithm and 2.5 mm dose grid.

	s25	s50	s80	s25–s80	p
MU/Gy	558 ± 101	463 ± 45	375 ± 41	-33%	0.04
MLC average aperture [cm]	1.3 ± 0.3	1.6 ± 0.4	1.9 ± 0.6	+39%	0.004
Modulation Index MI	20.5 ± 3.2	18.2 ± 3.5	15.1 ± 2.2	-26%	0.006

### Pre-treatment verification

**Figure 6:**
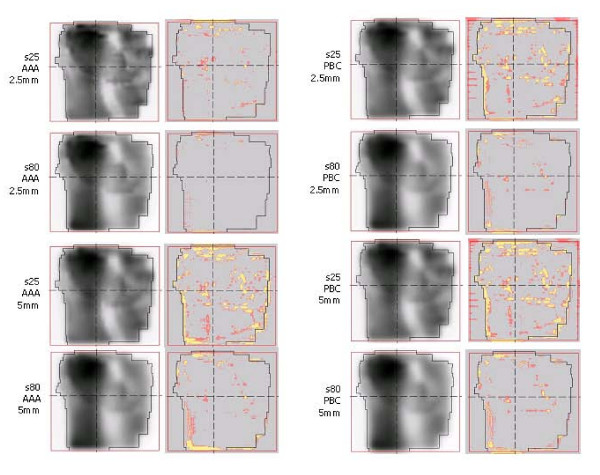
Example of the pre-treatment verification analysis. First column: calculated dose matrix. Second column: colour coded *γ *matrix (grey: *γ *< 1, pink: 1 <*γ *< 1.5, yellow: *γ *> 1.5).

Figure 6 presents, for one field, examples of the pre-treatment verification analysis. For the two dose calculation algorithms and for the two dose grid resolutions data are shown for the s25 and s80 experiments. The first column presents the calculated dose matrix, the second column the colour coded *γ *matrix (grey means *γ *< 1, pink 1 <*γ *< 1.5 and yellow *γ *> 1.5), Measurements and calculations are performed at the depth of d_max _= 1.5 cm in water.

Qualitatively it is evident how the accuracy of delivery is strongly affected by all three components: fluence complexity, calculation algorithm and dose grid resolution giving the best results for the combination: s80, AAA and 2.5 mm. For PBC the resolution of narrow peaks and valleys as well as the management of tails outside the modulated field area is compromised.

To quantify the agreement between calculated and measured dose maps and to correlate it with *MI*, values of %FA (averaged over all the fields) have been reported in table 4 for all the smoothing levels, algorithms, and grid resolutions. All the differences in table 4 are statistically significant, with p < 0.01 for all cases (except for s80, 5 mm grid and AAA against s80, 5 mm grid and PBC, where p = 0.07). The correlation coefficients between *MI *and %FA are reported, too. Those coefficients decrease with *MI *and with the dose calculation grid, and the lowest value is obtained for s80, AAA and 2.5 mm grid. This trend suggests that, when correlation is low, %FA is dominated by the real delivery issues, being the dose calculation engine settings properly selected to reproduce the expected fluence modulation. On the contrary, a high correlation could suggest that poor reliability in delivery is possibly generated by an excessive degree of modulation, and small changes in *MI *could influence directly the quality of the delivery.

**Table 4:** Summary of the pre-treatment verification analysis in terms of %FA, averaged over all the 17 fields for the different configurations of smoothing parameters, dose calculation algorithm and grid. MI values and the correlation coefficient between MI and %FA are also reported.

	s25		s50		s80	
MI	20.5 ± 3.2		18.2 ± 3.5		15.1 ± 2.2	

	%FA	correlation	%FA	correlation	%FA	correlation

AAA, 2.5 mm	6.4 ± 3.0	0.569	5.5 ± 2.2	0.398	2.7 ± 1.4	0.235
AAA, 5.0 mm	15.0 ± 5.7	0.787	10.8 ± 4.1	0.753	6.7 ± 2.3	0.692
PBC, 2.5 mm	15.3 ± 6.1	0.706	10.9 ± 4.3	0.621	5.6 ± 1.8	0.363
PBC, 5.0 mm	18.0 ± 6.6	0.729	15.1 ± 4.6	0.688	7.3 ± 2.0	0.563

**Figure 7:**
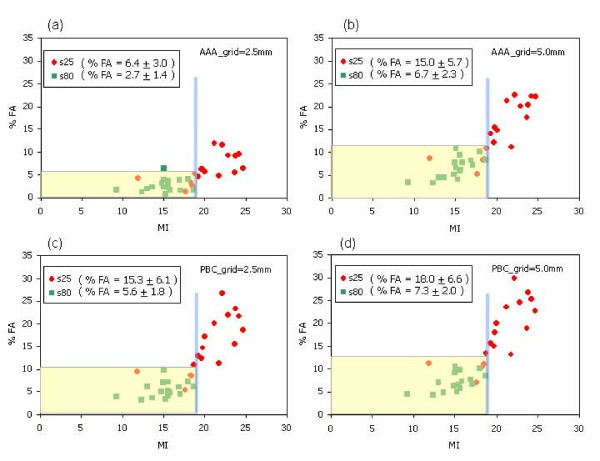
Scatter plot of the correlation between MI and %FA for some configurations. As a hatched band, the %FA achievable at 95% confidence level when a threshold of MI = 19 is applied is also shown.

Figure 7 presents scatter plots of %FA vs MI. For all the s80 data, the maximum observed value of MI is 19. Fixing therefore a threshold at MI = 19 (the vertical line in the graphs) and combining all the data from s25, s50 and s80 experiments, the resulting %FA, at 95% confidence interval, is: 5.5% for AAA-2.5 mm, 11.5% for AAA-5 mm, 10.0% for PBC-2.5 mm and 12.5% for PBC-5 mm. Fixing MI = 19, the probability to have %FA < 5% would be: 90%, 40%, 60% and 22% respectively. These results suggest that, with this value of MI, only for the case of AAA and 2.5 mm it would be possible to guarantee a satisfactory agreement between calculation and measurement.

With larger dataset (collected from routine application of these methods), it would be probably possible to determine the maximum MI value that could lead, at 95% confidence level, to a measured %FA < 5%. In this case MI could be used as a truly predictive indicator of the quality of the entire IMRT chain at a very early stage of the process (ideally already after optimal fluence calculation).

## Discussion and Conclusion

The study summarised in this report aimed to investigate possible correlations between the complexity of intensity fluences in IMRT treatment planning, measured by MI, and a variety of indicators related to dose plan quality, delivery efficiency and delivery accuracy.

The strategy of minimising fluence complexity without compromising plan quality, as suggested by Webb [18] was here followed, and a good predictive metric was looked for, in order to appraise possible features of an IMRT treatment at a very early stage of its planning procedure.

Excessive modulation leads to high numbers of MUs necessary to deliver prescribed doses with potential consequences on long term effects as secondary cancer induction [31], on treatment time for individual fractions (possibly to relate to organ movement and biological issues) and on radiation protection items.

In conclusion, it can be summarised, with a reasonably degree of generality, that:

• MI can be used as a reliable parameter to test different approaches/algorithms to smooth fluences implemented in a TPS, and to identify the preferable default values for the smoothing parameters if appropriate tools are implemented. A MI threshold set at MI < 19 could ensure that the planned beams are safely and accurately delivered within stringent quality criteria. The proposed threshold is likely numerically valid for the Varian environment, but it suggests an operational strategy for further applications.

• A reduction in fluence complexity is strictly correlated to a corresponding reduction in MUs, as well as to an increase of the average sliding window width (for dynamic IMRT delivery).

• A smoother fluence results in a reduction of dose in the healthy tissue with a potentially relevant clinical benefit.

• increasing the smoothing parameter s, MI decreases with %FA: fluence complexity has a significant impact on the accuracy of delivery;

• fixing a dose calculation grid, the photon dose calculation algorithm has an important impact on a better agreement between calculation and delivery, being more reliable the more advanced as intuitively expected.

• fixing a dose calculation algorithm, the finer the dose calculation grid, the better the agreement with delivery.

The considerations expressed above, intuitive in nature, allow to quantify, even with some restriction to their general value, the effect that shall be expected when insufficient computational means are adopted in IMRT. In particular the dose calculation with too simple algorithms has a remarkable impact in the disagreement between expected and actually delivered dose distributions. The issue of spatial resolution has an interplay with the previous argument and suggests also that insufficient algorithms remain defective also with finer resolutions while, with advanced algorithms, care shall be put in making available sufficient computational power to avoid compromises due to speed limits.

Some considerations shall be added to clarify some limitations of the present study.

It was carried out on data generated from a single TPS implementing only the concept of sliding window delivery. It would be important to expand the investigation to other environments and to the step-and-shoot method based on "patchwork" sequence of static fields or on the arc modulated techniques like Helical Tomotherapy or IMAT. Similarly, the study was limited only to two dose calculation algorithms, even if representing the two classes of algorithms as introduced by Knöös [32] and to one method only for managing fluence smoothness. In this respect the inclusion of a lung case would have been of interest to further appraise the behaviour of AAA in the presence of light tissues but in our clinic, IMRT is not applied to lung patients. Further studies could incorporate this area too.

The analysis was limited to physical dose quantities and to technical aspects of treatment delivery, without investigating any impact of fluence complexity on biological indicators and therefore did not address the correlation between degree of modulation and dose-painting, tumour dynamics, treatment delivery time. Analysis on the basis of the Equivalend Uniform Dose (EUD) estimator could be a first approach to biological appraisal.

With the present analysis we investigated aspects related to the capability of linear accelerators and multileaf collimators to reliably deliver complicate dose patterns, and to the time needed to deliver a given modulation pattern and to the number of monitor units necessary to the purpose.

The timing problem could be further related to radiobiological issues like the intra-fraction interplay between delivery time and cellular repair time [33]. The MU problem obviously relates to radiation protections issues and the risk of secondary cancer induction [31]. The impact of high fluence complexity on the IMRT efficacy can be investigated also in terms of clinical effects, even under the assumption of proper delivery. Bortfeld [1], addressing the interplay effects between intra-fractional organ's movement and fluence complexity proved that, even if in general organs' movement induce averaging effects, the presence of narrow fluence peaks or valleys, small body displacements could lead to severe local over-dosages or under-dosages. Excessive complexity in the fluence could have a negative trade-off also against inter-fraction tumour dynamics (e.g. hypoxic conditions, tumour stem cells migration, etc) that could be incorporated in the planning strategies [34]. These considerations should be linked to a rather old but still valid note of caution published by Goitein and Niemierko [35] where they proved the principle that the risk of treatment failure is more linked to dose deficits (severe under-dosages to small volumes) rather than to small/moderate under-dosages to larger volumes.

A final concern that could raise from this study is the possibility to determine a "proper" or "necessary" amount of modulation necessary to obtain an high quality plan. This can be hardly achieved by any study since it is obvious that, visible from the data shown here, the trade-off between fluence complexity, delivery issues and clinical implication would prevent any a-priori rules.

The main conclusion of our investigation is that tools can be easily developed to ascertain if a given set of fluences, generating a clinically acceptable plan, would have negative implications at delivery level with fast, semi-automatic numerical methods.

The availability in the TPS of the computation of the two-dimensional Modulation Index could therefore significantly minimise the need of pre-treatment measurements and solve, pragmatically, the question if "sufficient" or "excessive" modulation is introduced giving deeper insights of the IMRT chain.

## Competing interests

The author(s) declare that they have no competing interests.

## Authors' contributions

Study design: GN, AF, LC.

Data collection, analysis and development of methods: AC, EV, AF, GN.

DICOM software interfaces: FA.

Manuscript writing: LC.

Manuscript Review and final approval: all authors
